# The White Ceiling Heuristic and the Underestimation of Asian-American Income

**DOI:** 10.1371/journal.pone.0108732

**Published:** 2014-09-30

**Authors:** Chris C. Martin, John B. Nezlek

**Affiliations:** 1 Emory University, Atlanta, Georgia, United States of America; 2 College of William & Mary, Williamsburg, Virginia, United States of America; 3 University of Social Sciences and Humanities, Poznan, Poland; University of Vienna, Austria

## Abstract

The belief that ethnic majorities dominate ethnic minorities informs research on intergroup processes. This belief can lead to the social heuristic that the ethnic majority sets an upper limit that minority groups cannot surpass, but this possibility has not received much attention. In three studies of perceived income, we examined how this heuristic, which we term *the White ceiling heuristic* leads people to inaccurately estimate the income of a minority group that surpasses the majority. We found that Asian Americans, whose median income has surpassed White median income for nearly three decades, are still perceived as making less than Whites, with the least accurate estimations being made by people who strongly believe that Whites are privileged. In contrast, income estimates for other minorities were fairly accurate. Thus, perceptions of minorities are shaped both by stereotype content and a heuristic.

## Introduction

The belief that ethnic majorities dominate ethnic minorities has been supported by considerable research on intergroup processes. Scholars have labeled this belief the majority–minority paradigm [1] or minority disadvantage [2]. In the North American context, this belief has also been called White privilege [3], and has been promoted in higher-education pedagogy [4], [5]. Nevertheless, there are also instances in which ethnic minorities dominate a majority or surpass a majority in some indicator of status [6].

Given this state of affairs, how do people perceive members of an ethnic minority that does not conform to stereotypes held about minorities? It is possible that people rely on an aggregate stereotype, but it is also possible that people rely on a social heuristic that minorities cannot surpass the majority, which we term the *White ceiling heuristic* in the American context. Perceptions of groups may reflect a combination of both stereotypical thinking and the operation of the heuristic. The present study examined these possibilities by focusing on lay perceptions of the incomes of different ethnic groups in the US, and by analyzing these perceptions in terms of stereotypic and heuristic thinking.

### Stereotypic and Heuristic Information

No single definition of stereotype has been consistently used by social psychologists, but the scholarly consensus is that “stereotypes involve ascribing characteristics to different social groups or segments of society” [7]. According to the Evaluation–Potency–Activation (EPA) model, stereotypes exist in the mind, and their activation hinges on the potency (or latency) of the stereotype [8]. Stereotypes vary in the evaluative dimension (positive–negative), such that stereotypes can be anywhere on a spectrum between highly favorable and highly unfavorable. Stereotypes vary in terms of accuracy, some stereotypes being more accurate than others [7]. And stereotypes vary in terms of whether they are modeled at the population level or individual level. For instance, if American adults, on average, believe that the typical income of professors is $50,000, this constitutes a population-level stereotype about professors. This type of stereotype is termed a consensual or cultural stereotype in the EPA model. Of course, referring to such a belief as a stereotype only makes sense if the distribution of estimates is normal, and the standard deviation of estimates is reasonably low. These two conditions are typically satisfied. If an individual holds the belief that the typical income of professors is $50,000, this constitutes an individual-level stereotype about professors. This type of stereotype is termed a personal stereotype [7]. Cultural stereotypes are typically modeled by averaging the personal stereotypes of a sample. In the current study, we focused on cultural stereotypes about race (or ethnicity) and income. Our primary focus was on the accuracy of such stereotypes, although we were also concerned with the evaluative dimension, which we discuss later. We did not focus on potency.

Much psychological research on stereotypes (and the related topics of prejudice and discrimination) has focused on irrational negative stereotypes that powerful groups hold about subordinate groups [7]. Thus, the study of stereotypes has become intertwined with the study of hierarchical relationships, with only a small group of social psychologists focusing on stereotype accuracy [9]–[11]. This neglect of stereotype accuracy may also arise from skepticism about whether accuracy can be quantitatively modeled and whether accuracy can ever be achieved. Regardless, such concerns have been comprehensively rebutted by research which has established ways of measuring accuracy and demonstrated that stereotypes vary in their accuracy [10].

Within the American context, social scientists who use the hierarchical paradigm sometimes group all ethnic minorities together and term them *people of color* [12]–[16]. This label does not necessarily indicate that non-White subgroups are homogeneous; rather, it reflects a common experience of White racism. Nevertheless, the inclusive term *people of color* can be misleading because it suggests that these subgroups share some common attributes, even though category membership depends upon the *lack* of an attribute (i.e., Whiteness). Because the term *people of color* is inclusive rather than exclusive, it can lead to the perception that non-Whites are a well-defined group of people who are disadvantaged relative to Whites. Even though this bipartite division permits the acknowledgment of numerous inter-group differences among non-Whites, it also suggests that Whites dominate non-Whites, and that Whites may set the ceiling on any measure of success [17]. Thus, the use of the term *people of color* may make it more difficult to perceive heterogeneity on dimensions such as income, where Whites are both above and below non-White subgroups.

Given the dominance of this hierarchical paradigm during the past century—both within scholarship and everyday life [12], [16]—the paradigm has likely permeated the American consciousness. As a result, Americans might not only hold stereotypic beliefs about each ethnic group, but also perceive that a stable racial hierarchy underlies society, in which Whites outrank all ethnic minorities. Because hierarchies are easily detected and remembered [18], people may consequently create and apply a heuristic whereby Whites are seen as generally outranking non-Whites.

The current study examined whether this heuristic exists, and whether it is relevant to perceptions of income. We gathered lay estimates of the incomes of four ethnic groups that are commonly distinguished: Asian, Black, Hispanic, and non-Hispanic White. Using the mean difference of estimated and actual incomes, we examined if Asian-American income, which anomalously surpasses White income, is subject to significant underestimation in accordance with a White ceiling heuristic.

### Asian-Americans and Income

We focused on Asian-American income because Asian-Americans are an anomalous ethnic minority. Along with other ethnic minorities, Asian Americans were victims of severe discrimination in the past [19]–[21]. This discrimination reached a nadir during the Second World War when Japanese Americans were imprisoned in concentration camps [22]. Nevertheless, unlike most other American ethnic minorities, Asian Americans have surpassed White Americans in two important socioeconomic indicators: education and income. The average education level of Asian Americans is higher than that of Whites, and median Asian-American income surpassed White income about three decades ago [23], [24].

Income heterogeneity exists among Asian-Americans. Consider the median incomes of its six largest subcategories, who comprise approximately 83% of the Asian-American population: Indian ($88,000), Filipino ($75,000), Japanese ($65,390), Chinese ($65,050), Vietnamese ($53,400), and Korean ($50,000) [25]. Two of these subgroups have median incomes that are marginally lower than White American income ($56,178). Nevertheless, the incomes of Asian-Americans as a collective surpass White median income.

One could argue that the Asian American anomaly only indicates a trivial shift in the racial hierarchy, given that education and income levels refer to merely one group on merely two dimensions. This counterargument fails because Asians are not merely one group among a hundred, but rather one group among three, the other two being Hispanics and Blacks. (We exclude Native Americans because they are mostly residentially segregated from the rest of the populace.) Given the “denominator” of three, an increase in the “numerator” from zero to one constitutes a dramatic shift. Furthermore, education and income are not marginal social indicators, but rather primary indicators of socioeconomic status (SES), a major factor in social research. In addition, this change is not ephemeral. As noted above, Asian income surpassed White income approximately three decades ago and has consistently remained higher [23], [24].

The reasons for Asian-American socioeconomic success involve manifold sociological and psychological factors. In the labor market, wage discrimination against Asian-American men has ended [29]. Culturally, Asian Americans, particularly Asian-American men, are driven by the cultural norm of high educational achievement [30], [31]. Psychologically, stereotype lift enhances Asian performance in domains where Asians are positively stereotyped [32], [33]. Stereotype lift is the converse of stereotype threat, which is the phenomenon of negative stereotypes triggering anxiety among targets of the stereotype. These factors likely converge to raise Asian-American income and education levels, causing people to perceive Asians as a model minority, a stereotype that is controversial but sociologically accurate in some respects [1], [34].

Some criticism has been leveled at attempts to show the accuracy of this stereotype. To falsify the stereotype, some social scientists have pointed out that Hmong Asians have a particularly low median income, and thus one should not treat Asians as generally well off. In evaluating such suggestions, it is important to note that Hmong Asians constitute only 4% of Asian-Americans. The White population can also be divided ethnically into subgroups, and some of these (e.g., the Amish) have low median incomes [1].

Researchers who rely on the stereotype content model (SCM) also claim that Asians are victims of negative prejudice. According to the SCM, warmth and competence are the two primary dimensions on which stereotypes vary. In this two-dimensional framework, Asians are typically evaluated by White Americans as high in competence but low in warmth. Although SCM researchers have claimed these findings indicate motivated prejudice against Asians, such a claim neglects the differences in Western and Eastern conceptions of happiness. The Western conception involves positive affect (i.e., effusive warmth), whereas the Eastern conception involves balanced affect (i.e., equanimity) [35]. From a Western perspective, East Asians may therefore seem insufficiently warm, but this is a cultural misinterpretation, not an instance of invidious prejudice. Similarly, SCM research shows that Asians are perceived to be high in *foreignness* [36], but SCM research researchers have neglected the fact that the biggest waves of immigration from Asia occurred in the past four decades, whereas Americans of African and European descent arrived much earlier. Any subgroup whose arrival has been relatively recent may seem more foreign, and this perception may not constitute invidious prejudice.

Others have noted that Asians continue to be victimized through discriminatory treatment. Some evidence shows this is true [37], but all groups including Whites experience some degree of negative discrimination [38], and Asians also benefit from positive discrimination [33]. In addition, victimization neither confirms nor falsifies the model minority stereotype, because such information is orthogonal to the stereotype’s content. We do not claim that society should be indifferent to the unique problems faced by the Asian-American community, nor do we claim that Asians are always on the positive end of social disparities. We do claim that Asians have an anomalously positive standing in the socioeconomic domain, and if people assume a strict racial hierarchy, such standing may go unnoticed.

### Stereotype Accuracy and Income

Stereotypes are commonly portrayed as inaccurate in the psychological literature, but empirical studies of accuracy have shown that cultural stereotypes tend to concur with objective data, and the effect sizes of accuracy research are among the strongest in social psychology [10]. Over 20 studies have examined accuracy in stereotypes about race, ethnicity, sex, occupation, college major, nationality, and political ideology [7]. To quantify accuracy, data from participants were tested via correlations or discrepancy scores against population data from the U.S. Census or similar sources. High levels of accuracy were found for group-level stereotypes, and moderately high levels were found for person-level stereotypes. For instance, women’s socioeconomic progress over the past decades is accurately represented in participants’ retrospective, contemporaneous, and prospective perceptions of women’s salaries [39]. It is worth noting that SCM researchers are generally unconcerned with accuracy, whereas EPA researchers consider accuracy to be a primary attribute of stereotypes.

Given the general accuracy of cultural stereotypes, participants in the current study who rely solely on stereotypes should accurately estimate income for different ethnic groups. Participants who rely solely on stereotypes should estimate Asian income as higher than White income. In contrast, if estimates of income reflect the operation of the White ceiling heuristic, participants should report Whites as having higher income than other ethnic groups. Given the reality that Asian-Americans make more money than Whites, believing that Whites make more than Asians requires grossly underestimating Asian incomes perhaps in combination with overestimating White incomes. If stereotypes and the proposed heuristic combine, participants’ perceptions will vary based on the target group. When accurate information about a group accords with the White ceiling heuristic, as in the case of Blacks and Hispanics, the agreement between the two will reinforce the reliance upon accurate information. When accurate information about a group conflicts with the heuristic, as in the case of Asian Americans, participants will use cognitive algebra [40] to arrive at an intermediate point between the stereotype and the heuristic.) For Asian-American targets, the conflict between the two types of information should produce an estimate of Asian Americans lagging slightly behind Whites.

We caution that our use of the term *stereotype* here to denote a specific reasoning process doesn’t indicate that stereotypic reasoning is always distinct from heuristic thinking. We simply use the pragmatic terms *stereotype* and *heuristic* to describe two contrasting processes in a readable manner.

Some may counter-argue that if Whites under-estimate Asians, this phenomenon may reflect aversive prejudice, but on empirical grounds, this counter-argument seems weak. White prejudice against Asian Americans seems to be minimal. (The first author, who is an Asian immigrant and has lived in the American South for 19 years, can attest to this experience in his own life.) In a recent cross-national survey [25], only a small percentage (13%) of Asian Americans viewed discrimination against their group as a major problem. When asked about whether their Asian identity makes a difference when it comes to gaining admission into colleges, finding a job, and getting a promotion, the percentage of respondents who stated that their identity would help matched or surpassed the percentages who stated it would hurt. In addition, approximately 60% of participants stated that their identity would neither help nor hurt them. Furthermore, 91% reported that they get along “very well” or “pretty well” with Whites. In addition, intergroup contact and cooperation occur frequently, as manifested by the extraordinary rate of intermarriage with other races and the low rate of Asian-American residential segregation [25], [41]. Even laboratory research shows that Whites implicitly believe Asians are less dangerous than Whites themselves [42], but we believe field research makes a more compelling case because of its greater external validity.

Nevertheless, some aversive prejudice against Asians may cause people to perceive Asians as having lower incomes than Whites. To measure whether individual differences in intergroup prejudice cause estimates of Asian income to lag behind White income, we measured social dominance orientation (SDO) in the current study. As we discuss later, SDO was not correlated with estimates of the Asian–White gap, which suggests that intergroup prejudice does not manifest itself in a manner that concerns us.

The over-estimation of Whites may not seem to have the same implications as, say, the under-estimation of minorities, but the majority–minority model rests upon the assumption that Whites can be unambiguously ranked at the top of the racial hierarchy. As a result, social scientists often rely on the majority–minority model by prescribing the acknowledgement of White Privilege. The current study suggests that people who rely on this model may have beliefs that are not factually justified.

Among social scientists, the White privilege model may persist because Asians are often excluded from study samples, leading to comparisons between Whites, Blacks, and Hispanics exclusively [43], [44]. The economist Thomas Sowell has suggested that statistics about Asians are excluded from reports about non-White minorities to maintain the simplistic heuristic of using raw statistics as evidence of discrimination. For instance Sowell notes, “There has been much indignant outcry in the media when statistics have shown that black applicants for mortgage loans were turned down more often than white applicants.... Yet statistical data on Asian Americans have been conspicuous by their absence.... If such data are included, it turns out that, in 2000, black applicants were turned down for primary mortgage loans twice as often as white applicants—and white applicants were turned down nearly twice as often as Asian American applicants” [6]. Similarly, the sociologist Arthur Sakamoto and his colleagues have noted that “instructors of Asian American Studies courses will invariably encounter the ‘Are we minorities?’ question from Asian American undergraduates. The typical answer is that Asian Americans are a sociological minority that is often not officially classified as a minority because their socioeconomic attainments are not significantly lower than those of whites” [1]. A search of articles in the database PsycInfo reveals that between 2000 and 2014, only 140 publications contained both “stereotypes” and “Asian Americans,” whereas 646 contained both “stereotypes” and “African Americans.” And the American Sociological Review has “apparently never published a paper focusing on the educational attainment or incomes of Asian Americans” [1]. Although the study of Blacks’ underprivileged status is critical to evaluate stratification in America, the exclusion of Asians can unwittingly bias conclusions about Whites’ status. Problematically, the application of the White ceiling heuristic may also cause people to overlook the positive attainments of ethnic minorities, a lack of recognition that cyclically maintains the heuristic’s superficial validity. Although publicized minority achievements in the U.S., such as those of African Americans in basketball and Asian Americans in spelling bees, are likely to be remembered, unpublicized achievements may go unnoticed.

### Hypotheses of the Current Study

In the current study, we expected to find that estimates of incomes reflect a cognitive algebra: accuracy dominates perceptions of income, but this accuracy is reduced by the White ceiling heuristic. Focusing on income, a primary socioeconomic indicator, we hypothesized that lay estimates for “typical” minorities (i.e., Blacks and Hispanics) would be accurate, whereas lay estimates pertaining to Asian Americans would be uniquely inaccurate. As representations of a tempered stereotype, they should be comparable to estimates of Whites, but fall slightly behind the White median. As inaccurate stereotypes, they should evince uniquely low accuracy.

On an exploratory basis, we also tested a set of auxiliary hypotheses. Our first auxiliary hypothesis was that people who were better educated or had higher incomes would be more knowledgeable about Asian trends due to homophily and greater familiarity with Asians, and would estimate incomes more accurately than more poorly educated or lower-income individuals. We therefore measured income and education. Second, as noted earlier, we hypothesized that social dominance orientation (SDO), being an antecedent of xenophobic prejudice [45], might motivate negative perceptions of Asians [46]. In particular, Whites who are high in SDO may be motivated to perceive Asians as lagging behind Whites, a White–SDO interaction. Finally, we hypothesized that Asians themselves would make accurate estimations of the White–Asian difference, given that homophily drives people to become more familiar with members of their own race. For exploratory reasons, we also measured gender, age, political orientation (conservative vs. liberal), and subjective confidence in one’s estimates.

## General Method

### Ethics Statement

Participants gave their written informed consent, and the protocol was approved by the Protection of Human Subjects Committee at the College of William and Mary.

### Participants

Participants of all three studies were adult U.S. residents who used Mechanical Turk [47], [48]. Their demographic characteristics are summarized in Table 1. Mechanical Turk is an online labor market managed by Amazon.com, where digital tasks such as surveys can be administered. Each person who completes the task (i.e., each survey participant) is paid a fixed sum of money. Because the participant pool on Mechanical Turk is much more diverse than the typical convenience sample [49], Mechanical Turk has become a popular tool for behavioral research [50], [51]. In our studies, each participant was paid 25 cents (Study 1), 75 cents (Study 2), or 5 cents (Study 3).

**Table 1:** Participant Demographics.

	Study 1	Study 2
Age	18–67	18–73
Range	27.5	29
Median	31	32.8
Mean		
Gender	64%	53%
Male	35%	46%
Female		
Income	$0–$200,000	$1,000–$600,000
Range	$45,000	$43,000
Median	$51,888	$56,722
Mean		
Ethnicity	4.9%	4.1%
Black	79.9%	80.9%
White	6.6%	5.5%
East Asian	4.2%	2.3%
Hispanic	1.7%	3.2%
Biracial/Multiracial		
Education		
High School or Less	8.7%	12.7%
Some College	39.9%	31.8%
College Degree	39.6%	44.5%
Masters Degree	8.7%	7.7%
Doctoral Degree	2.4%	3.2%
N	286	213

*Note*. Demographic data were not collected in Study 3.

### Procedure

Participants were asked to make their best estimate of the median household income of each major ethnic group (i.e., Asian, Black, Hispanic, and White) and U.S. residents overall in studies 1, 2, and 3. In Studies 1 and 2, participants also estimated the percentage of people living in poverty among each ethnic group and among US residents overall. Participants were informed that a four-person household is poor if the household income is less than $24,500; and an adult living alone is poor if the adult earns less than $10,250. This definition of poverty was presented to participants after they entered their estimates of median household income. Refer to Appendix S1 for income and poverty questions.

Participants entered all incomes on the same screen—they were aware of the differences between their estimates. Thus, the difference scores computed from these estimates represent real perceptions of income gaps, rather than artificially derived difference scores that are computed for statistical convenience [52]–[54].

Political orientation was measured using questions about party preference, economic attitudes and social attitudes (α = .85 to .86) [55]. The first question was “How would you describe your political party preference?” and participants answered on a scale from 1 (*strong* Republican) to 7 (*strong Democrat*). The second question was “In term of economic issues, how would you describe your political attitudes and beliefs?” Participants answered on a scale from 1 (*very conservative*) to 7 (*very liberal*). The third question was “In terms of social issues, how would you describe your political attitude and beliefs?” Participants answered on a scale from 1 (*very conservative*) to 7 (*very liberal*).

SDO was measured using a four-item scale (α = .79 in Studies 1 and 2) [55]. Participants were asked to state whether they had a positive or negative feeling towards these four statements: “In setting priorities, we must consider all groups,” “We should not push for group equality,” “Group equality should be our ideal,” and “Superior groups should dominate inferior groups.” The second and third statements were reverse scored. Participants answered on a scale from 1 (*very negative*) to 7 (*very positive*).

In studies 1 and 2, we found that SDO, the SDO × White interaction term, political orientation, education, income, gender, race, and age were unrelated to the dependent variable, and we removed the corresponding measures in Study 3.

Based on *a priori* guidelines for deciding what responses were valid, we deleted some submissions. Submissions were deleted if any of these conditions were met: (a) the participant finished the survey in less than two minutes; (b) the participant computed the poverty rate for any group as zero; (c) the participant put all group estimates above or below their estimate for national median; (d) the participant estimated the median income of a group as less than $10,000 or greater than $150,000; or (e) the participant implicitly estimated the ratio between any two group incomes as greater than 10∶1. Duplicate submissions from individual participants in the same study were deleted. Ten participants participated in both Study 1 and Study 2. We deleted their submissions in Study 2, because one of the goals of Study 2 was to replicate the results of Study 1.

### Data Analysis

We measured two types of inaccuracy of judgments, *underestimation* and *mis-estimation*. To measure *underestimation*, we subtracted a participant’s income estimate from the accurate figure for that group. To measure *mis-estimation*, we took the absolute values of the raw inaccuracy scores. Each of these measures was analyzed with a repeated-measures ANOVA that allowed us to compare inaccuracy of judgments for different groups. Planned contrasts were used to compare the inaccuracy score for Asians to the mean inaccuracy score for the other three target groups combined. Effects sizes for contrasts were computed by calculating the mean difference score (representing the contrast of interest), and dividing this score by its standard deviation [56].

### Significance Testing

**Figure 1:**
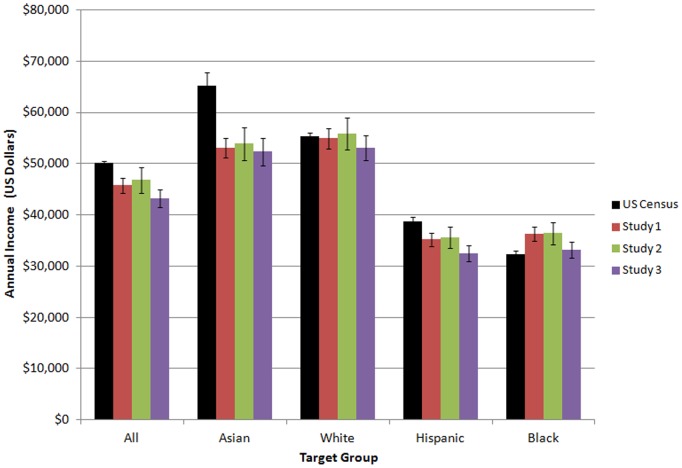
U.S. Census Bureau figures for median income and mean lay estimates of median income. Error bars denote the 90% confidence interval for Census figures, and the 95% confidence interval other figures. The Census figure for White median income represents non-Hispanic Whites.

**Figure 2:**
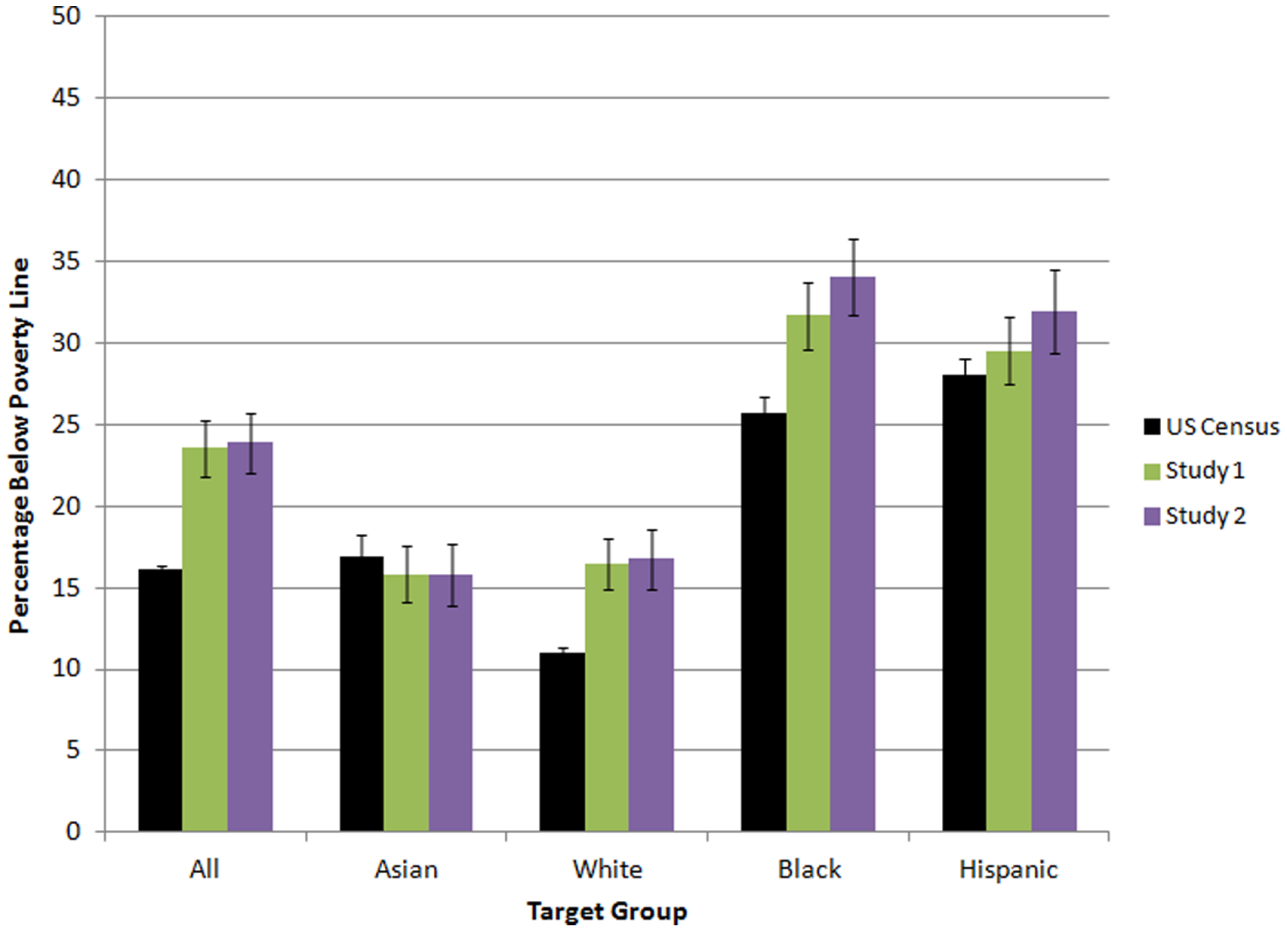
U.S. Census Bureau figures and mean estimates of poverty percentages. Error bars denote the 90% confidence interval for Census figures, and the 95% confidence interval for other figures. The U.S. Census figure for Whites represents non-Hispanic Whites.

Because we collected data on five target groups we could have conducted significance tests to measure absolute inaccuracy by target group, relative inaccuracy by target group, rank-based accuracy across all groups, and planned contrasts for all combinations of groups. We did not conduct this battery of tests because our hypotheses suggested specific comparisons. Moreover, in terms of avoiding Type I error, we replicated our findings across the three studies, as shown in Figures 1 and 2. We felt that our findings’ robustness was well established by such replication. Readers may find it more useful to refer to these figures than to the p values in the significance tests.

## Study 1

The purpose of the first study was to gather lay estimates of income and poverty to determine if people accurately estimate inter-group differences in poverty and income.

### Participants

Data from 288 participants were retained from a total of 359 unique submissions.

### Method

The procedure described in the General Method section was used.

### Results and Discussion

Participants’ estimates for income and poverty are shown in Figures 1 and 2, alongside accurate figures from the U.S. Census Bureau [23], [57] that we use as a benchmark for accuracy. Repeated-measures ANOVAs revealed a significant main effect of target group in under-estimation, *F*(3,861) = 154.4, *p*<.001 and mis-estimation, *F*(3,861) = 70.3, *p*<.001. The planned contrast for Asians was statistically significant for both underestimation, *F*(1,287) = 296.2, *p*<.001, *d* = −1.01 and mis-estimation, *F*(1,287) = 117.6, *p*<.001, *d* = 0.64. Thus, Asian income was uniquely underestimated and mis-estimated.

Overall U.S. poverty was dramatically overestimated (see Figure 2), possibly because data were collected when unemployment levels were high. Analyses by target group revealed that poverty was quite accurately estimated for Asians, which suggests an acknowledgement of Asian economic success. In terms of the focus of our paper, the White ceiling heuristic would have been falsified only if White poverty had been estimated as greater than Asian poverty. This did not occur however. Estimates of Asian and White poverty did not differ significantly, *t*(287) = 1.5, *p* = .13.

**Figure 3:**
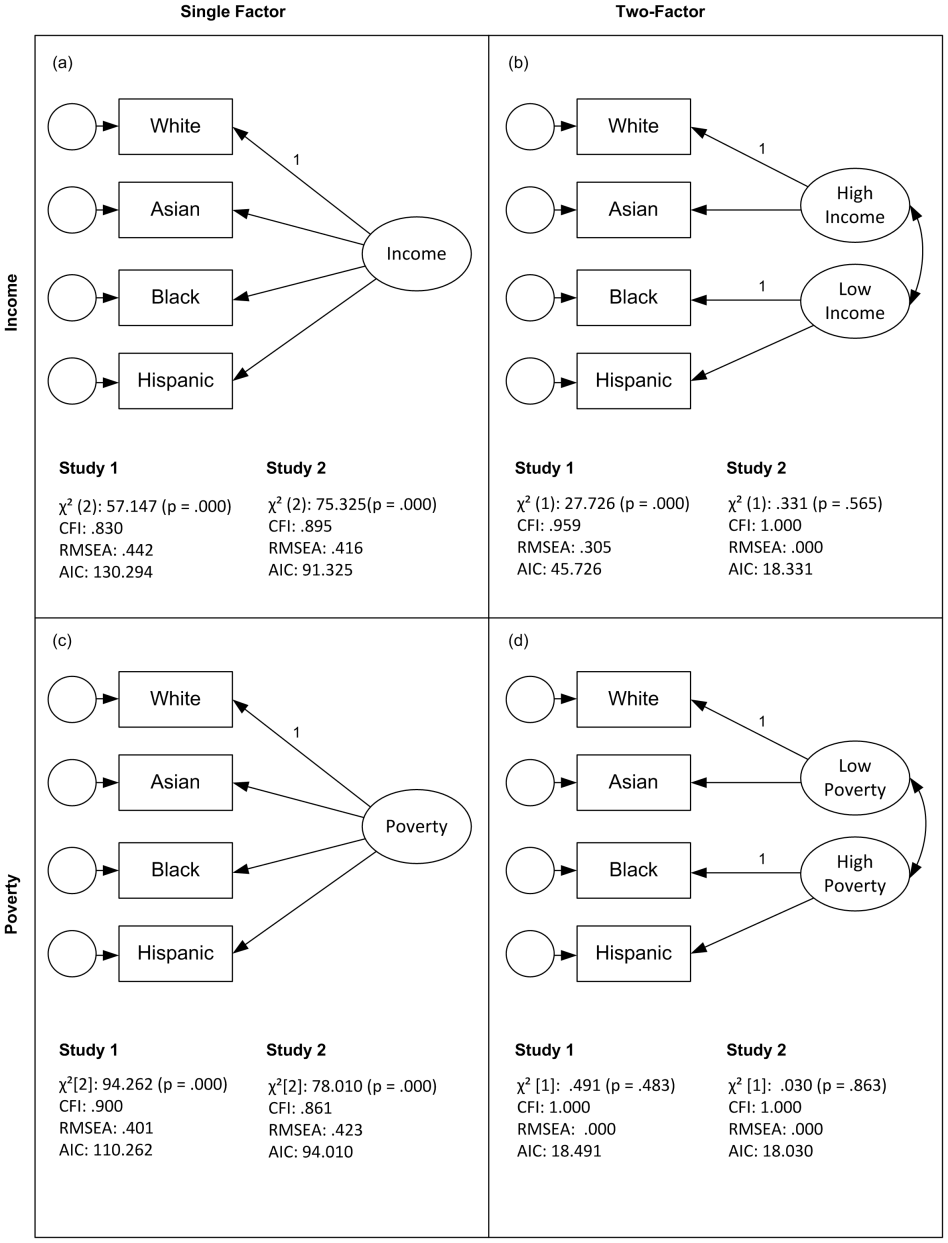
Confirmatory factor analyses of estimated income and poverty. CFI = comparative fit index. RMSEA = root mean square error of approximation. AIC = Akaike information criteria. BIC = Bayesian information criteria.

Estimates of income were positively inter-correlated (*r* range = .51–.84), as were estimates of poverty (*r* range = .68–.84) (Tables 2 and 3). Given these correlations, we conducted confirmatory factor analyses (CFA) to test if people made estimates based on a single implicit anchor of general income (or poverty), or based on two implicit anchors: one for higher-income (low poverty) groups, namely, Whites and Asians, and the second for lower-income (high poverty) groups, namely, Hispanics and Blacks. We predicted that the two-factor solution would have better fit, and our prediction was supported for both income and poverty (Figure 3). Despite their underestimation of Asian income, participants accurately perceived Asians and Whites as members of the same higher-income cluster.

**Table 2:** Correlations Between Estimates of Income in Study 1.

	All	White	Black	Asian	Hispanic
All	1				
White	.84***	1			
Black	.78***	.64***	1		
Asian	.76***	.72***	.52***	1	
Hispanic	.69***	.51***	.78***	.56***	1

*Note*. ***p<.001.

**Table 3:** Correlations Between Estimates of Poverty in Study 1.

	All	White	Black	Asian	Hispanic
All					
White	.81***				
Black	.71***	.70***			
Asian	.71***	.83***	.68***		
Hispanic	.71***	.71***	.84***	.70***	

*Note*. ***p<.001.

Although estimates of Black and Hispanic were almost identical, the estimate for Black income was relatively higher than estimated Hispanic income by a small margin. Yet actual Black median income lags behind Hispanic median income. Participants may have erred in these estimates because of the availability heuristic: A layperson might easily recall wealthy Black persons in politics, entertainment, and sports. Blacks, having arrived in the U.S. before the major Hispanic immigration wave, may also appear to be a more established minority. This explanation is purely speculative, however, and the Black–Hispanic discrepancy was so small that it may be meaningless.

To examine individual differences in inaccuracy of estimated incomes, we conducted a hierarchical regression with Asian–White difference as the dependent variable (Table 4). One of the predictors was participant race, which we limited to two dummy-codes, one representing if a participant was White and another indicating if a participant was Asian. We modeled only these two ethnic categories because the Asian-White difference was the focus of our study. We also entered SDO (social dominance orientation) and the interaction of SDO and White ethnicity as predictors to examine if the effect of SDO varied between Whites and non-Whites. The other predictors were estimated White income, confidence in one’s answer, education level, income, gender, age, and political orientation.

**Table 4:** Hierarchical Multiple Regression Analyses Predicting Asian–White Income Difference in Study 1 (N = 234).

	Model 1			Model 2			Model 3		
Variable	B	SE B	*β*	B	SE B	*β*	B	SE B	*β*
Constant	15034.1	2456.6		16089.2	6148.6		18774.0	7775.4	
Estimated White income	−0.31	0.04	−0.43^***^	−0.32	0.04	−0.44^***^	−0.33	0.05	−0.46^***^
Confidence in answer				−529.1	918.2	−0.03	−736.3	946.8	−0.05
Education Level				−511.4	886.3	−0.04	−680.1	908.5	−0.05
Income				0.03	0.02	0.08	0.03	0.02	0.10
Gender				1764.5	1566.6	0.07	1827.2	1615.8	0.07
Age				−75.2	66.6	−0.07	−75.2	69.0	−0.07
SDO							−515.4	1492.3	−0.05
Political Orientation							623.3	598.5	0.08
White							−3559.5	4711.4	−0.11
Asian							−4250.2	4117.7	−0.07
White × SDO							745.7	1678.8	0.08
R^2^		0.18			0.01			0.01	
*F* for change in R^2^		51.43^***^			0.80			0.50	

*Note*. *** = p<0.001. White and Asian are dummy coded. The White × SDO interaction term was entered to examine if SDO specifically predicted estimation among Whites. Political Orientation was scored such that higher scores indicated greater liberalism.

The only significant predictor was estimated White income, *B* = −.31, *SE*_B_ = .04, β = −.43, *p*<.001, which suggests that participants relied on a ratio (rather than a fixed difference) to estimate Asian income after estimating White income. If participants had relied on a fixed difference (e.g., estimating Asian income to be $500 lower than White income regardless of absolute White income), this coefficient would have been non-significant. When estimated White income was removed, the other coefficients remained non-significant. Using G*Power 3.1.7, we found that the post hoc statistical power of this regression was .99 for an effect size of .15. Contrary to our expectations, SDO and the SDO × White interaction term were non-significant predictors of the Asian–White difference.

## Study 2

In Study 2, we sought to replicate our findings and examine participants’ subjective reaction to accurate income figures. We anticipated unique surprise at an accurate report of Asian income, indicating that the figure meaningfully exceeded expectations.

### Participants

Data from 213 participants were retained from a total of 245 submissions.

### Method

All of the measures used in Study 1 were used in Study 2. In addition, we showed participants accurate income and poverty figures after participants entered income estimates. Participants were asked whether they were surprised by these figures. They reported surprise on a five-point scale ranging from 1 (*not at all surprising*) to 5 (*completely surprising*). Participants who answered 2 or greater were directed to a question in which they checked reasons for their surprise. Their options were “Overall people are better/worse off than I thought,” “Whites are better/worse off than I thought,” etc. For each pair, the statement pertaining to being worse off was coded as −1, better off as 1, and no answer as 0.

### Results and Discussion

The findings from Study 1 were replicated. Repeated-measures ANOVAs produced a significant main effect of target group in under-estimation, *F*(3,636) = 80.5, *p*<0.001 and mis-estimation, *F*(3,636) = 47.8, *p*<.001. The planned contrast for Asians was statistically significant for underestimation, *F*(1,212) = 193.5, *p*<.001, *d* = −0.95 and mis-estimation, *F* (1,212) = 89.4, p<.001, d = 0.65. Again, Asian income was uniquely underestimated and mis-estimated.

The correlations and CFA results were also replicated, as shown in Figure 3 and Tables 5 and 6. Again, only estimated White median income predicted Asian–White Difference, *B* = −.19, *SE*_B_ = .04, β = −.36, *p*<.001 (Table 7). When estimated White income was removed, the other coefficients remained non-significant. A post hoc analysis revealed the statistical power was .99 for an effect size of .15.

**Table 5:** Correlations Between Estimates of Income in Study 2.

	All	White	Black	Asian	Hispanic
All					
White	.90***				
Black	.82***	.69***			
Asian	.84***	.83***	.76***		
Hispanic	.77***	.63***	.83***	.71***	

*Note*. ***p<.001.

**Table 6:** Correlations Between Estimates of Poverty in Study 2.

	All	White	Black	Asian	Hispanic
All					
White	.72***				
Black	.69***	.61***			
Asian	.66***	.76***	.64***		
Hispanic	.67***	.55***	.82***	.58***	

*Note*. ***p<.001.

**Table 7:** Hierarchical Multiple Regression Analyses Predicting Asian–White Income Difference in Study 2(N = 187).

	Model 1			Model 2			Model 3		
Variable	B	SE B	*β*	B	SE B	*β*	B	SE B	*β*
Constant	8097.5	2280.7		6869.2	6575.4		7680.5	7539.5	
Estimated White income	−0.20	0.04	−0.36^***^	−0.20	0.04	−0.36^***^	−0.20	0.04	−0.36^***^
Confidence in answer				588.6	961.6	0.04	668.5	995.2	0.05
Education Level				1174.0	1012.5	0.08	1273.4	1052.4	0.09
Income				−0.02	0.01	−0.11	−0.02	0.01	−0.12
Gender				1130.9	933.4	0.09	1109.4	986.1	0.09
Age				−18.0	81.9	−0.02	−20.3	84.3	−0.02
SDO							63.5	1242.5	0.01
Political Orientation							−423.1	691.6	−0.05
White							1115.5	2920.9	0.06
Asian							−33.0	2371.2	0.00
White × SDO							−839.8	1169.5	−0.12
R^2^		0.13			0.02			0.01	
*F* for change in R^2^		25.77***			0.97			0.24	

*Note*. *** = p<0.001. White and Asian are dummy coded. The White × SDO interaction term was entered to examine if SDO specifically predicted estimation among Whites. Political Orientation was scored such that higher scores indicated greater liberalism.

Next, we examined subjective surprise at accurate income figures. After viewing actual income and poverty figures, 21.1% of participants found the figures *not at all surprising*, and 78.9% expressed some level of surprise between *slightly surprising* and *completely surprising*. A repeated-measures ANOVA that compared answers on the follow-up questions regarding whether surprise was triggered by the target group being better off or worse off than expected produced a significant main effect of target group, *F* (3,480) = 7.38, *p*<.001. A planned contrast was used to compare the mean surprise level for Asians (*M* = .24, *SD* = .72) against the combined mean for Whites (*M* = .10, *SD* = .60), Blacks (*M* = −.06, *SD* = .62), and Hispanics (*M* = .01, *SD* = .74). The contrast was significant, *F*(1,160) = 13.95, *p* = .001, *d* = 0.29. The contrast was also significant when the surprise level for Asians was compared against the combined mean of only Blacks and Hispanics, *F*(1, 160) = 15.78, *p*<.001, *d* = 0.31. Thus, participants were uniquely surprised about Asian socioeconomic status being higher than they expected.

## Study 3

In this study, we examined if people tend to underestimate Asians relative to Whites because they adopt the White ceiling heuristic, thus assuming all minorities lag behind Whites in social indicators. Instead of measuring the heuristic in abstract terms, we used a White-privilege measure designed for an U.S. context. Participants reported how strongly they believed that Whites had a privileged status in the U.S. They reported their *belief* that Whites were privileged, not their endorsement of White privilege, which was implicitly measured by SDO in studies 1 and 2. Thus, items were constructed to measure individual differences in whether participants believed that American society conferred advantages on Whites without measuring individual differences in sympathy toward non-White groups. Thus, participants who believed that Whites were highly privileged would obtain a high score on this scale, regardless of whether they endorsed or resented such privileges. We hypothesized that belief in the existence of White privilege would predict White–Asian difference.

### Participants

We retained data from 192 participants out of 225 unique submissions.

### Method

Participants entered their estimates of income as in the previous studies. Estimates of poverty were not collected.

We used a four-item scale to measure belief in the existence of White privilege (which for brevity we henceforth term *Belief in White Privilege).* Scales of White privilege found in the literature had two flaws: they were constructed to be administered only to Whites, and they contained emotionally loaded items. We therefore constructed a new scale, and measured Belief in White privilege using four questions, each answered on a 7-point scale and prefixed with “In the United States.” The questions were: “Do you think people treat Whites worse or better than non-Whites in general?”, “Do you think Whites need to work less hard or work harder than non-Whites to get ahead in their career?”, “Do you think Whites have fewer opportunities or more opportunities than non-Whites”, and “Do you think Whites have fewer advantages or more advantages in life than non-Whites?” Appendix S2 contains the full scale with all scale anchors.

Answers were averaged and midpoint-centered to compute each participant’s score (α = .86). Midpoint-centering was used so that positive scores would indicate that the respondent believed Whites are privileged, negative scores would indicate that the respondent believed non-Whites are privileged, and zero would indicate neutrality.

### Results and Discussion

As in the prior studies, repeated-measures ANOVAs produced a significant main effect of target group in under-estimation, *F*(3,573) = 70.3, *p*<.001, and mis-estimation, *F*(3,573) = 61.3, p<.001. The planned contrast for Asians was statistically significant for underestimation, *F*(1,191) = 136.5, *p*<.001, *d* = 0.84 and mis-estimation, *F*(1,191) = 106.8, *p*<.001, *d* = 0.74. Again, Asian income was uniquely underestimated and mis-estimated. Across the three studies, the average effect size was -.93 for underestimation and .68 for mis-estimation. By psychological standards, effect sizes of .5 and over are considered large [58].

The mean score on the Belief in White Privilege scale was .90 (*SD = *1.15, skewness = −0.55, kurtosis = 0.60). This mean score .90 was significantly greater than zero, *t*(191) = 10.84, *p*<.001, *d* = 1.57, indicating that participants, on average, rated Whites as privileged. In all, 14.1% of participants scored less than zero, 10.9% scored zero, and 75.0% scored greater than zero.

To examine the association between Belief-in-White-Privilege scores and magnitude of underestimation, we computed White–Asian Difference (as opposed to Asian–White difference) to make regression slopes easier to interpret. Using a hierarchical regression with White–Asian Difference as the dependent variable, we entered estimated White median income in Step 1, *R* ^2^ = .07, *F* = 13.26, *df* = 190, *p*<.001, and it was a significant predictor of White–Asian Difference, *B* = .188, *SE*_B_ = .05, β = .26, *p*<.001. We entered Belief-in-White-Privilege score in Step 2, *R* ^2^ *_change_* = .55, *F_change_* = 11.83, *df* = 189, *p* = .001. It was a significant predictor of White–Asian Difference, *B* = 2550.17, *SE*_B_ = 741.52, β = .24, *p* = .001. As hypothesized, people who rated Whites as highly privileged also estimated that White median income surpassed Asian-American median income by a substantial margin.

Belief in White privilege also predicted estimates favoring Whites for White–Black Difference, *B* = 1319.50, *SE*_B_ = 600.26, β = .10, *p* = .03, and White–Hispanic Difference, *B* = 1824.15, *SE*_B_ = 625.40, β = .14, *p* = .004. It should be noted, however, that White, Black, and Hispanic income were estimated fairly accurately, as shown in Figure 1. Belief in White privilege was not significantly correlated with estimates of White, Black, and Asian incomes (all *p*s>.11); and was negatively correlated with estimated Hispanic income, *r*(192) = −.155, *p* = .03.

The goal of Study 3 was to test the hypothesis that Belief in White Privilege was related to underestimation of the White-Asian income difference, and we therefore only included scales pertaining to this hypothesis. As a result, we cannot directly measure the discriminant validity of the Belief-in-White-Privilege to show that it differs from scales of prejudice. Regardless, the relationship between SDO and White-Asian underestimation was not significant (and not close to significant) in studies 1 and 2. In contrast, the relationship between Belief in White Privilege and Asian underestimation was highly statistically significant in Study 3. This difference cannot be attributed to greater statistical power because the sample sizes were comparable, suggesting that prejudice (as indexed by SDO) is probably distinct from Belief in White Privilege.

## General Discussion

Asian-American income was estimated just below White income in all of our studies. As hypothesized, it was also uniquely underestimated. This underestimation occurred across the social, political, and economic spectrum of respondents. Neither highly educated respondents nor highly confident respondents made estimates that were more accurate than the average respondent. Even respondents under 25, who have never lived in an era of superior White income, made incorrect estimates. The only factor that predicted mis-estimation was the belief that Whites are privileged, with intensity of belief associated with greater inaccuracy. Thus, the White ceiling heuristic seems to mislead all Americans, but the effect seems stronger among people who believe Whites are privileged.

Our findings suggest that Asian underestimation occurs even among the highly educated, which may include academic social scientists. In fact, the authors of a recent study about affirmative action explicitly claimed that “Whites earn more [than other groups]” [59]. Nonetheless, amidst the inaccuracy in our studies there was accuracy of a higher-order. The grouping of Asians and Whites into a high-income, low-poverty cluster indicated that Asians are accurately perceived as high earners. If the people-of-color concept is part of the collective consciousness, causing Blacks and Hispanics to be clustered, Asians clearly fall outside this cluster. This mixture of accuracy and inaccuracy was precisely what we predicted.

Given the tendency for participants to estimate majority income as higher than that of all subgroups, participants may perceive majority income as the ceiling under which all other subgroup incomes must fall. This perception likely arises from the implicit adoption of the White ceiling heuristic when people estimate inter-group resource distribution.

The adoption of a hierarchical racial paradigm may seem intuitive, given the history of inter-group relations in the U.S. Yet facts about contemporary U.S. society do not reveal a clear hierarchy, but rather an ambiguous stratification of ethnic groups. For instance, both Asians and Jews have education and income levels that put them at the top of a racial hierarchy, but both Asians and Jews are almost exclusively victims rather than perpetrators of hate crimes which seems to place them at the bottom of the same hierarchy [38], [60]. On the other hand, Blacks have the second highest per capita rate of victimization in hate crimes (after Jews), which seems to place them at the bottom, but Blacks also commit hate crimes at the highest per capita rate, which seems to place them at the top [38], [60]. Additionally, black men are perceived as more attractive than white men [61]–[63], but they are also perceived as more dangerous than white men [42]. Blacks and Asians also have better aggregate mental health than Whites [64]–[68], while Hispanics have better physical health than Whites [69]. The White ceiling heuristic may thus lead to invalid inferences about social strata.

The White ceiling heuristic not only has implications for perceptions about ethnic groups but also perceptions about societal units such as states and countries, units that people implicitly arrange in a hierarchy. For instance, people probably know that the U.S. sits at the top of the international hierarchy of wealth and military power. People may therefore infer that the U.S. also has the most prosperous citizens, even though per capita GDP, income equality, and life expectancy are higher elsewhere [70]. These false inferences, which stem from a ceiling heuristic, may drive attitudes toward governmental policies. Correcting such false assumptions would seem to be critical to having informed citizens.

### Conclusions and Limitations

The current results suggest that people’s perceptions of the incomes of US citizens are the result of at least two influences that sometimes oppose each other. The first is the reality of income differences. On average, members of some ethnic groups earn more money than members of other groups, and people’s perceptions of income appear to be informed by this reality. The second influence is what we have called the “White ceiling heuristic”—the extent to which people believe in a rigid hierarchy of social groups in a society. When these two influences suggest the same conclusion (e.g., perception of White income as higher than most minorities), the resulting point judgment is reasonably accurate. When they suggest different conclusions (i.e., estimates of Asian-Americans’ incomes), it appears that the heuristic combines with reality to produce a biased judgment.

These conclusions need to be considered within the context of the present study’s limitations. Although the available data suggest that Mechanical Turk samples are more representative of the American public than university-student samples and convenience samples [49], our samples were not explicitly designed to be representative. Moreover, our samples did not contain a large number of non-White participants. Given the increasing diversity of the U.S., more attention should be given to non-White participants in future work on this heuristic. Our measure of White privilege, although face valid and reliable, may not have measured the construct as well as intended. It did not capture distinct facets in participants’ assessment of privilege. We also did not measure explicit attitudes about ethnic groups, which may have been informative. It is also not clear the extent to which perceptions of income are similar to perceptions of other characteristics such as appropriateness for elected office. Perhaps most important, our results are limited to American society as it now exists. Whether a similar heuristic operates in other cultures remains to be seen.

Nevertheless, we believe that the present results make a meaningful contribution to our understanding of intergroup relations, stereotype accuracy, and perceived income inequality. Social hierarchies are ubiquitously perceived, and a thorough understanding of heuristics and biases needs to take the influence of such hierarchies into account.

## Supporting Information

Appendix S1 **Measures of Income, Poverty, and Confidence in One’s Answer.**
(DOCX)

Appendix S2 **Measure of Belief in White Privilege.**
(DOCX)

Appendix S3 **Datasets.**
(ZIP)
