# Correction: The White Ceiling Heuristic and the Underestimation of Asian-American Income

**DOI:** 10.1371/journal.pone.0116228

**Published:** 2014-12-17

**Authors:** The PLOS ONE Staff

There are errors in Table 1. Each value in the second and third columns between rows 1 and 17 should be shifted down by one row. Please see the corrected Table 1 here.

**Table 1:** Participant Demographics.

	Study 1	Study 2
Age		
Range	18–67	18–73
Median	27.5	29
Mean	31	32.8
Gender		
Male	64%	53%
Female	35%	46%
Income		
Range	$0–$200,000	$1,000–$600,000
Median	$45,000	$43,000
Mean	$51,888	$56,722
Ethnicity		
Black	4.9%	4.1%
White	79.9%	80.9%
East Asian	6.6%	5.5%
Hispanic	4.2%	2.3%
Biracial/Multiracial	1.7%	3.2%
Education		
High School or Less	8.7%	12.7%
Some College	39.9%	31.8%
College Degree	39.6%	44.5%
Masters Degree	8.7%	7.7%
Doctoral Degree	2.4%	3.2%
N	286	213

*Note*. Demographic data were not collected in Study 3.
