# Supplementary material for: The White Ceiling Heuristic and the Underestimation of Asian-American Income
Source: PLoS One. 2014 Sep 30;9(9):e108732. doi: 10.1371/journal.pone.0108732 (PMC4182537; doi:10.1371/journal.pone.0108732)
Supplement: Appendix S1 — Measures of Income, Poverty, and Confidence in One’s Answer. (DOCX) [file pone.0108732.s001.docx]

**Appendix S1. Measures of Income, Poverty, and Confidence in One’s Answer**

Some of the following questions are about median household income, which is a statistic. The median income is the ³middle´ income for a group of people. Half the people in a group make less than the median, and half make more. So, the median can be thought of as the typical income.

Please make sure that you understand the meaning of "median." Then proceed.

What do you think the median yearly income is for the following types of households in the United States? Household refers to both families and to individuals who live alone.

Enter your estimate in the box next to each label (rounded to the nearest thousand). Do NOT put a $ sign before each answer. (If you choose not to answer, put -1 in the box.)

Median Household Income (US Dollars)

All Households _____

White Households _____

Black Households _____

Asian Households _____

Hispanic Households _____

How confident are you in your answer to this question?

Totally confident

Highly confident

Moderately confident

Slightly confident

Not confident at all

I choose not to answer

The following question is about how many people live in poverty in the U.S. Per the U.S. government definition, a family with two adults and two children is poor if they earn less than $24,500; and an adult living alone is poor if he or she earns less than $10,250. How many people in the US in each of the following groups do you think lives in poverty? Please use a percent

from 0 (no one in that group lives in poverty) to 100 (everyone in that group lives in poverty).

(If you choose not to answer, put -1 in the box.)

All Households _____

White Households _____

Black Households _____

Asian Households _____

Hispanic Households _____

How confident are you in your answer to this question?

Totally confident

Highly confident

Moderately confident

Slightly confident

Not confident at all

I choose not to answer
