# Supplementary material for: The White Ceiling Heuristic and the Underestimation of Asian-American Income
Source: PLoS One. 2014 Sep 30;9(9):e108732. doi: 10.1371/journal.pone.0108732 (PMC4182537; doi:10.1371/journal.pone.0108732)
Supplement: Appendix S2 — Measure of Belief in White Privilege. (DOCX) [file pone.0108732.s002.docx]

**Appendix S2. Measure of Belief in White Privilege**

1. In the United States, do you think people treat Whites worse or better than non-Whites in general?

Much worse

Worse

Somewhat worse

About the same

Somewhat better

Better

Much Better

2. In the U.S., do you think Whites have fewer opportuniteis or more opportunities than non-Whites?

Whites have much fewer opportunities

Whites have somewhat fewer opportunities

Whites have slightly fewer opportunities

Whites have the same opportunities

Whites have slightly more opportunities

Whites have somewhat more opportunities

Whites have many more opportunities

3. In the U.S., do you think Whites have fewer advantages or more advantages in life than no-Whites?

Whites have much fewer advantages

Whites have somewhat fewer advantages

Whites have slightly fewer advantages

Whites have the same advantages

Whites have slightly more advantages

Whites have somewhat more advantages

Whites have many more advantages

4. In the U.S., do you think Whites need to work less hard or work harder than non-Whites to get ahead in their career?

Whites need to work much less hard

Whites need to work somewhat less hard

Whites need to work slightly less hard

Whites need to work the same

Whites need to work slightly harder

Whites need to work somewhat harder

Whites need to work much harder
